# Identifying priority countries for scaling up small-quantity lipid-based nutrient supplements

**DOI:** 10.1136/bmjgh-2025-019353

**Published:** 2025-12-11

**Authors:** Navideh Noori, Christine P Stewart, Christine M McDonald, Kimberly Ryan Wessells, Elisabeth D Root, Kathryn G Dewey

**Affiliations:** 1 Institute for Disease Modeling, Gates Foundation, Seattle, Washington, USA; 2 Institute for Global Nutrition, Department of Nutrition, University of California Davis, Davis, California, USA; 3 Departments of Pediatrics, and Epidemiology and Biostatistics, University of California San Francisco, San Francisco, California, USA

**Keywords:** Stunting, Prevention strategies, Nutrition, Child health

## Abstract

**Introduction:**

Undernutrition is a cause of nearly half of all deaths among children under 5 years old. Small-quantity lipid-based nutrient supplements (SQ-LNS) have been shown to prevent child wasting, stunting, anaemia and mortality among children 6–23 months of age in low- and middle-income countries (LMICs). Scaling up effective preventive interventions is urgent given the current global food insecurity and nutrition crisis.

**Method:**

To prioritise SQ-LNS scale-up activities, we identified countries with the highest burdens of wasting, stunting and all-cause mortality among children 6–23 months of age at the national level using the most recent national survey data including the Demographic and Health Survey and Multiple Indicator Cluster Surveys, as well as the Lives Saved Tool in LMICs. National-level estimates informed a care cascade model to assess the potential impact of SQ-LNS on all-cause mortality, stunting and wasting. We also conducted a subnational level analysis among the 20 highest burden countries with the most recent available survey data to identify the highest burden regions.

**Results:**

Our analysis identified the top 20 countries with the highest burden of the three outcomes as: Niger, South Sudan, Yemen, Sudan, Somalia, Democratic Republic of Congo, Eritrea, Nigeria, Central African Republic, Guinea, Equatorial Guinea, Chad, Papua New Guinea, Benin, Mali, Angola, Pakistan, Timor-Leste, Sierra Leone and Côte d'Ivoire, although for some countries the survey data were collected >10 years ago. Some of these countries also ranked high in population estimates of acute food insecurity. The care cascade model demonstrates that a large number of cases of stunting and wasting and deaths could be potentially averted if SQ-LNS is provided.

**Conclusion:**

Most of the top 20 countries are in Sub-Saharan Africa, with a few in South and Southeast Asia. This geographical concentration underscores the urgent need for targeted interventions in these regions to prevent child malnutrition.

**WHAT IS ALREADY KNOWN ON THIS TOPIC**

- It is known that small-quantity lipid-based nutrient supplements (SQ-LNS)—nutrient-dense pastes fortified with essential vitamins, minerals and fatty acids—reduces child wasting, stunting, anaemia and mortality among children 6–23 months of age in low- and middle-income countries.
- However, survey reports generally estimate stunting, wasting and mortality rates among all children under 5 years of age, and not specifically for the age group of 6–23 months old.

**WHAT THIS STUDY ADDS**

- By estimating the burden of stunting, wasting and all-cause mortality for the specific age group of 6-23 months old at the national and subnational level, and developing a care cascade model—a decision-tree simulation framework that applies SQ-LNS effect sizes to national burden estimates, we identified high burden countries and regions where SQ-LNS will be most impactful. No other study has focused on this age group even though it is a particularly vulnerable period for undernutrition.

**HOW THIS STUDY MIGHT AFFECT RESEARCH, PRACTICE OR POLICY**

- Our analysis could help decision-makers and funders to determine where scale-up of SQ-LNS should be prioritised.

## Introduction

 Undernutrition is a cause of nearly half of all deaths in children under 5 years of age.^1^ In 2024, stunting affected an estimated 23.2% or 150.2 million children under 5 years of age globally, and wasting affected 6.6% or 42.8 million.^2^ The Sustainable Development Goals (SDGs) target 2.2 calls for an end to all forms of malnutrition by 2025, with a 40% reduction in stunting (relative to 2012), and for wasting to occur in less than 5% of children.^3^ There is an urgent need to scale up effective actions to improve child nutrition, especially given the current nutrition crisis and ongoing conflicts around the world. However, only one out of three wasted children receives the care they need due to insufficient resources and an uncoordinated system.^4^ In addition to increasing funding for the early detection and treatment of child wasting, the prevention of wasting and other forms of child undernutrition should also be prioritised.^5^ However, there are limited effective strategies for the prevention of undernutrition.

Provision of small-quantity lipid-based nutrient supplements (SQ-LNS) is a relatively new preventive intervention that was added to the list of recommended interventions to effectively address child undernutrition^6^ and is one of the few interventions that can help to achieve multiple SDG targets concurrently.^7^ SQ-LNS are small packets of nutrient-dense paste that were designed to supplement the diets of children 6–23 months of age, a period when nutrient requirements are high, and diets may lack multiple micronutrients and essential fatty acids. Each packet contains more than 20 essential vitamins and minerals and essential fatty acids to support healthy growth and development. They have been shown to reduce child wasting, stunting, anaemia and mortality among children 6–23 months of age in low- and middle-income countries (LMICs).^7 8^ SQ-LNS is the only preventive intervention for children that has demonstrated a simultaneous beneficial impact on several outcomes in vulnerable populations.^5^ For certain outcomes, the greatest impact was seen in sites with higher burdens of stunting or wasting or poorer water quality or sanitation. The benefit-cost ratio of SQ-LNS was estimated to be 13.7 times when targeted to the children 6–23 months of age in the 60% of the population with the lowest socioeconomic status in the 40 LMICs with the highest prevalences of stunting.^9^ In its recent guidelines on (1) prevention and management of wasting and nutritional oedema (acute malnutrition) in infants and children under 5 years and (2) complementary feeding for infants and young children 6–23 months of age, the WHO recommended the use of SQ-LNS in food insecure populations.^10 11^ UNICEF also emphasises the importance of targeting the youngest children in the most vulnerable households and in food insecure settings with high burden of wasting and stunting and micronutrient deficiencies.^12^ Provision of SQ-LNS is not a stand-alone intervention but rather should be considered as part of a package of interventions and integrated into existing programmes and services including household food assistance in settings with high levels of food insecurity.^12 13^ Identifying the most vulnerable populations and targeting provision of SQ-LNS to these groups could optimise its impact.^7^ Despite strong evidence showing that SQ-LNS delivers consistent and significant benefits across multiple outcomes for children aged 6–23 months, uptake at scale remains limited in most countries. The goal of our work is to identify countries with a high burden of stunting, wasting and all-cause mortality among children aged 6–23 months old, for prioritising the settings where SQ-LNS will be most impactful. While many surveys report undernutrition data for all children under 5 years of age, this study focuses specifically on children 6–23 months old, a particularly vulnerable developmental window with elevated nutritional needs. By pinpointing where the burden is highest and where SQ-LNS would be most impactful, our study aims to support more efficient and equitable allocation of funding and programming resources.

Given that blanket and geographical targeting may be the most appropriate strategy for preventive programmes in high-risk populations,^14^ we also conducted a similar analysis at the subnational level for selected countries, using recent survey data to identify the highest burden regions within those countries.

## Methods

### National-level analysis

To identify priority countries for the scale-up of SQ-LNS, we estimated the prevalence of moderate and severe wasting and moderate and severe stunting and the all-cause mortality rate among children aged 6–23 months old at the national level in LMICs in Sub-Saharan Africa, South and Southeast Asia, as well as Middle East, North Africa, Central and South America regions, based on the World Bank classifications.^15^ These estimates were necessary since survey reports generally report stunting, wasting and mortality rates among all children under 5 years of age, and not specifically for the age group of 6–23 months old. Moderate stunting is defined as length-for-age z score (LAZ): −3≤ LAZ <−2, and severe stunting is defined as LAZ <−3. Moderate wasting is defined as weight-for-length z score (WLZ): −3 ≤ WLZ <−2, and severe wasting is defined as WLZ <−3.^16^

To estimate the moderate and severe stunting prevalence, we used survey estimates from the WHO Child Malnutrition database for 2021 among children aged 24–59 months old.^17^ We chose the age interval of 24–59 months to capture the total burden of stunting up to 24 months of age, since the stunting prevalence increases sharply during the age interval of 6–23 months. After 24 months, the prevalence of stunting generally stabilises, and the estimate for 24–59 months would be a good approximation of stunting that has occurred prior to 24 months of age. To derive the most recent estimates for each country, we developed a regression model between survey-based stunting estimates for children aged 24–59 months and the annual national-level Joint Malnutrition Estimate (JME) estimates^2^ for children under 5 years for years 2000–2021 to predict 2021 stunting prevalence among children aged 24–59 months. JME also reports annual estimates of wasting prevalence among children under 5 years old at the regional level. However, exploratory analyses indicated that there were no consistent trends in the prevalence of wasting across years that could be used to develop a regression model, as was done for stunting. Therefore, we used the most recent national survey data including the Demographic and Health Survey (DHS),^18^ Multiple Indicator Cluster Surveys (MICS)^19^ and WHO Child Malnutrition Estimates^17^ to estimate the prevalence of moderate and severe wasting among children 6–23 months of age. This is consistent with global trends, which are downward for stunting during the past 20 years, but not for wasting prevalence.

We estimated the all-cause mortality rate per 1000 live births among children 6–23 months of age from the Lives Saved Tool (LiST). LiST is a computer-based application for modelling the impact of maternal and child health interventions and calculates changes in cause-specific mortality based on intervention coverage change, intervention effectiveness for that cause and the percentage of cause-specific mortality sensitive to that intervention.^20^ LiST child mortality estimates are developed by the UN Inter-agency Group for Child Mortality Estimation (UN-IGME).^21^ UN-IGME estimates mortality by fitting a smooth trend curve over values from DHS, MICS, vital registration data and population census data.

After calculating prevalence estimates of moderate and severe wasting and moderate and severe stunting, as well as the all-cause mortality rate among children aged 6–23 months old, we ranked countries for each outcome. We then created a composite score by averaging the ranks across the following three outcomes: all-cause mortality rate, prevalence of severe wasting and prevalence of severe stunting. We also developed a separate composite score based on the all-cause mortality rate, the prevalence of stunting and the prevalence of wasting. We referred to the Integrated Food Security Phase Classification (IPC) scale for countries that rank high in acute food insecurity.^22^

**Figure 1:**
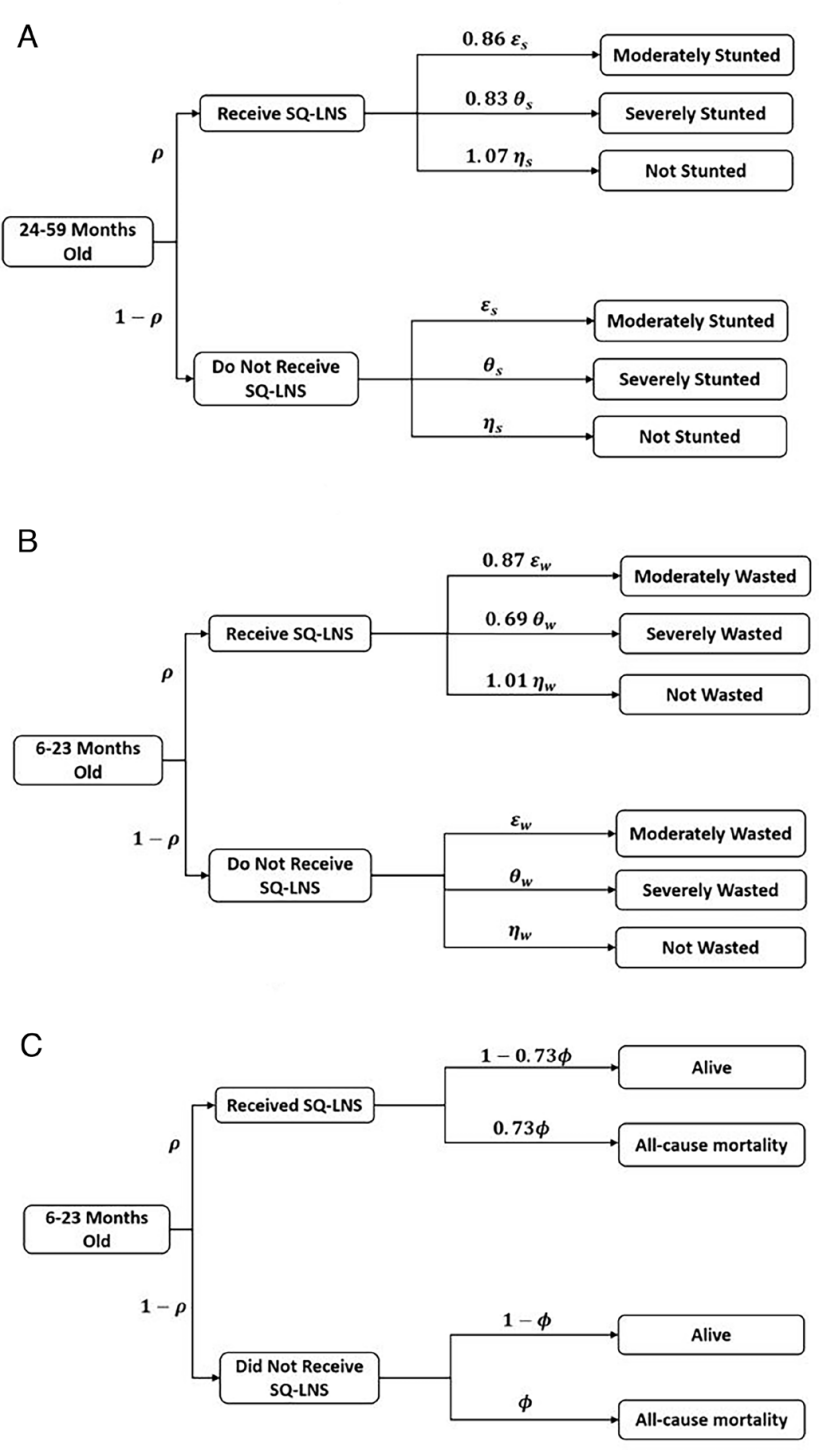
Schematic of decision tree model of (**a**) stunting, (**b**) wasting and (**c**) all-cause mortality. SQ-LNS, small-quantity lipid-based nutrient supplements.

We further developed a care cascade or decision tree model to estimate the potential effects of provision of SQ-LNS on reducing each outcome at the national level using the national-level prevalence estimates and mortality rate estimates. The decision tree model was developed for the top 20 countries based on the composite scores. We estimated the national-level population of children aged 6–23 months old by obtaining the population of children under 1 and 1–2 years old from the World Population Prospect.^23^ We divided the under-1 population by half, subtracted neonatal deaths (from UN-IGME) and deaths among 1–6 months old (~ 5* (infant deaths – neonatal deaths)/11) and then summed the result with the population of children aged 1–2 years old. We accounted for uncertainty in the simulation by employing Monte Carlo simulation techniques and defining a range of values for the prevalence and mortality estimates, as well as for relative reduction in risk of each outcome among those who receive SQ-LNS based on the meta-analysis of randomised controlled trials of SQ-LNS provided to children 6–23 months of age.^7 8 24^ For the prevalence estimates of wasting and stunting, we used CIs from the source of each estimate (DHS, MICS, WHO Child Malnutrition Database, WHO-JME predicted estimates), and for the relative reduction in risk of each outcome, we used the estimated CIs from the meta-analyses^7 8 24^ and generated a matrix of 1000 sample points for each outcome and relative reductions within the given range using a Latin Hypercube sample.^25^ The all-cause mortality estimates from LiST do not include CI.^7 8 22^ The percentage of children aged 6–23 months old who would receive SQ-LNS in the models varied between 0% and 100%. Descriptions of the model parameters and the decision model structure are given in table 1 and figure 1. All analyses were conducted using R.^26^

**Table 1:** Decision tree models parameters description

Parameter description	Parameter	Value
Percentage of children aged 6–23 months old who will receive SQ-LNS	ρ	0%–100%
Prevalence of moderate stunting and moderate wasting among children 6–23 months old	$\varepsilon_{s}$, $\varepsilon_{w}$	Baseline estimates
Prevalence of severe stunting and severe wasting among children 6–23 months old	$\theta_{s}$, $\theta_{w}$	Baseline estimates
Prevalence of children aged 6–23 months old who are not stunted and not wasted	$\eta_{s}=1-\varepsilon_{s}-\theta_{s}$ $\eta_{w}=1-\varepsilon_{w}-\theta_{s}$	Baseline estimates
All-cause mortality among children aged 6–23 months old	ϕ	Baseline estimates
Crude prevalence ratio of moderate stunting^7^	–	0.86 (0.83, 0.89)
Crude prevalence ratio of severe stunting^8^	–	0.83 (0.78, 0.90)
Crude prevalence ratio of moderate wasting^7^	–	0.87 (0.80, 0.95)
Crude prevalence ratio of severe wasting^8^	–	0.69 (0.55, 0.86)
Crude prevalence ratio of all-cause mortality^7^	–	0.73 (0.59, 0.89)

SQ-LNS, small-quantity lipid-based nutrient supplements.

### Subnational-level analysis

We conducted a similar analysis at the subnational level among the top 20 countries with the highest composite scores based on severe stunting, severe wasting and all-cause mortality with the most recent available survey data from DHS or MICS over the past 10 years to identify the highest burden regions within a country. A list of countries with recent survey data is given in online supplemental table S1.

## Results

### Heatmap analysis

**Figure 2:**
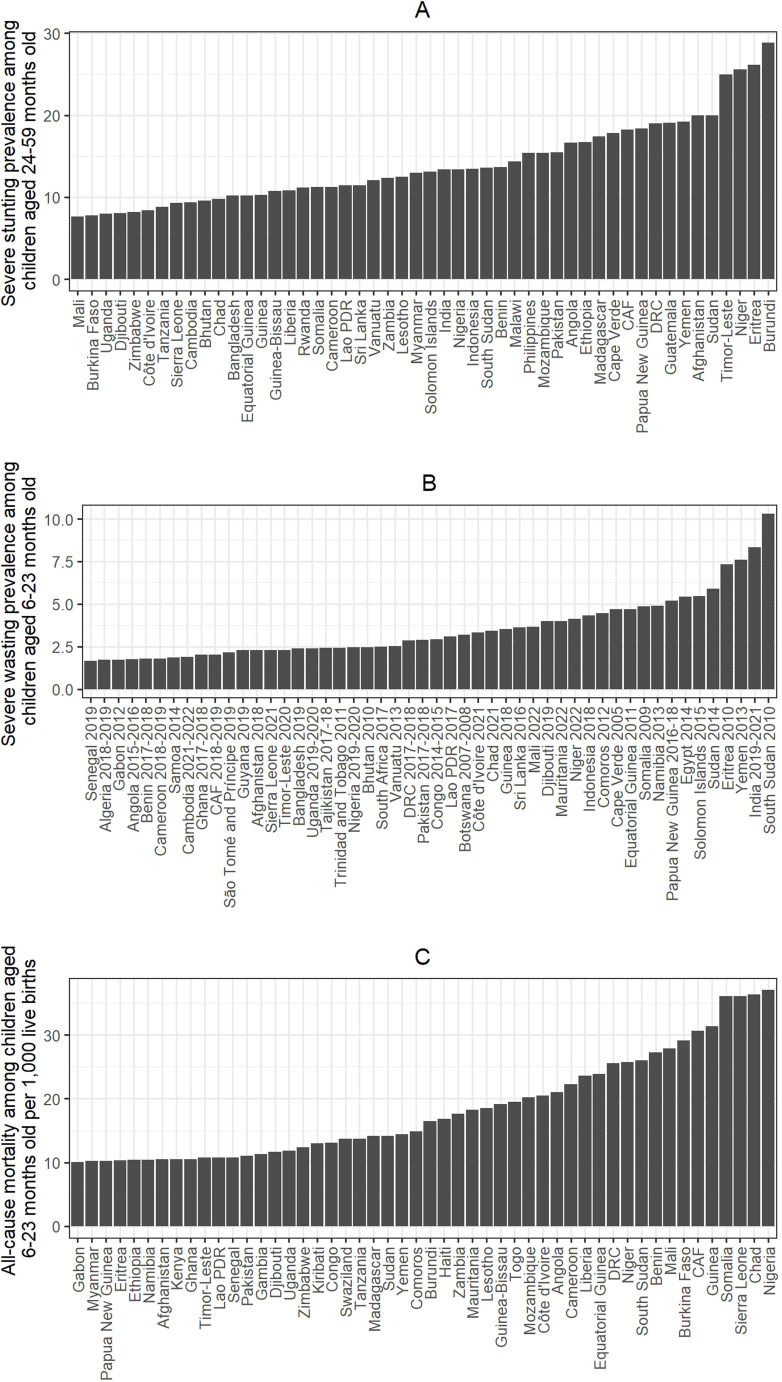
(**A**) Prevalence of severe stunting among children aged 24–59 months old for the year 2021, **(B**) prevalence of severe wasting among children aged 6–23 months old based on recent survey data and (C) all-cause mortality among children aged 6–23 months per 1000 live births for the year 2021. CAF, Central African Republic; DRC, Democratic Republic of the Congo; PDR, Lao People's Democratic Republic.

**Figure 3:**
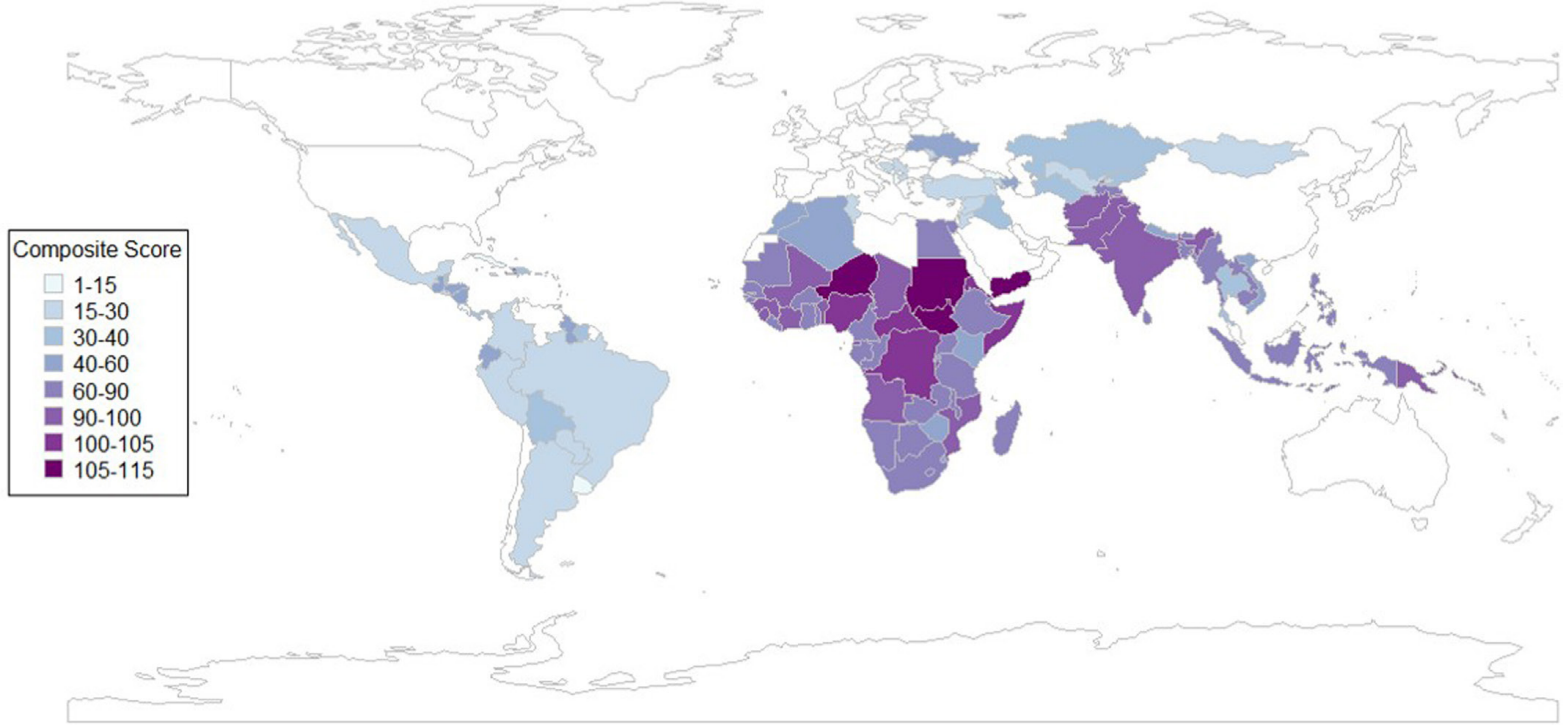
Heatmap of composite score of countries based on severe stunting, severe wasting and all-cause mortality.

The baseline prevalence estimates of severe wasting among children aged 6–23 months old based on the most recent survey data, prevalence estimates of severe stunting among children aged 24–59 months old for the year 2021, and the all-cause mortality rate among children aged 6–23 months per 1000 live births for 2021 from LiST are given in figure 2. For each outcome, the top 50 countries with the highest burden are shown. Estimates for other LMICs are shown in online supplemental figure S1. The y-axis for severe wasting shows the survey year used to estimate prevalence for each country. We ranked countries for each of these outcomes and averaged the ranks across the three outcomes. For each outcome, the ranking value ranges between 1 and 119 representing the number of countries included in this analysis. Figure 3 shows the heatmap of the composite score of countries and online supplemental figure S2 shows rankings in a bar graph. The composite score ranges between 1 and 115, and the higher the score is, the higher the mortality rate and the prevalence estimates of severe wasting and severe stunting. Niger, South Sudan, Yemen, Sudan and Somalia are the top five countries with the highest composite scores. The composite score we developed based on the mortality rate, prevalence of stunting and prevalence of wasting showed similar results (online supplemental figure S3–S5).

Our analysis suggests that 14 countries rank in the top 20 for at least two of the three outcomes: Niger, South Sudan, Yemen, Sudan, Somalia, Democratic Republic of Congo, Eritrea, Central African Republic, Guinea, Equatorial Guinea, Papua New Guinea, Benin, Mali and Angola, in rank order, although for some countries the survey data were collected over 10 years ago. Online supplemental figure S6 shows the countries with IPC phases 3 and higher for acute food insecurity in at least 20% of the country population. The x-axis in online supplemental figure S6 shows the most recent period for which IPC severity was estimated for each country. Among these countries, Yemen and South Sudan, with over 50% IPC phase 3 and above, experienced the highest acute food insecurity. The IPC rankings, however, focus on food insecurity often in countries facing humanitarian emergencies, whereas our rankings are based on mortality rates and the prevalence of child undernutrition among children 6–23 months old. Therefore, not all highly ranked countries in our analysis were ranked highly in IPC estimates.

### Decision tree model of potential effects of SQ-LNS on stunting, wasting and all-cause mortality

We estimated the potential downstream benefits of provision of SQ-LNS on the burden of stunting, wasting and all-cause mortality at the national level using the previously described decision tree model. We present the effect as the absolute numbers as well as percentage point reduction for each adverse outcome at the national level (online supplemental figures 7 and 8). Our simulation suggests that over 1.2 million cases of moderate stunting could be potentially averted if half of children aged 6–23 months old received SQ-LNS in the top 20 countries. Similarly, with 50% coverage, over 930 000 cases of severe stunting, over 200 000 cases of moderate wasting, over 165 000 cases of severe wasting and over 90 000 deaths could be potentially averted (table 2).

**Table 2:** Total number of cases for each outcome and potential number of cases of wasting and stunting and number of deaths averted, if half or all of children aged 6–23 months old in the 20 countries with the highest composite scores (based on prevalence of severe stunting, severe wasting and all-cause mortality) receive SQ-LNS

	Total number of cases for each outcome without provision of SQ-LNS	SQ-LNS coverage	
		50%	100%
Moderate wasting	3 440 339±11 668*	215 005±1834	430 011±3668
Severe wasting	1 120 618±2754	165 255±1198	330 510±2396
Moderate stunting	17 176 930±66 712	1 202 495±5772	2 404 990±11 545
Severe stunting	11 638 660±7521	931 033±4542	1 862 068±9085
All-cause mortality	718 416±0	93 393±695	186 787±1391

* mean±SE.

SQ-LNS, small-quantity lipid-based nutrient supplements.

### Subnational-level analysis

The full subnational rankings and prevalence estimates for severe wasting, severe stunting and mortality are provided in the online supplemental figures 9–36. These highlight the regions with the highest composite scores for each outcome within each country. Sudan, DRC and Nigeria are the highest burden countries with recent survey data. In Nigeria, using DHS 2018, the states of Kebbi, Sokoto and Katsina have the highest composite score with highest mortality rate and highest prevalence of severe stunting and severe wasting among children aged 6–23 months old. In DRC, using MICS 2017, Sankuru and Maniema are the highest burden provinces. In Sudan, using MICS 2014, the states of Gadarif, central and north Darfur have the highest burden of the three outcomes.

## Discussion

We identified countries with the highest burdens of stunting, wasting and all-cause mortality among children 6–23 months of age, with the objective of identifying candidate countries for the scale-up of SQ-LNS. Developing an approach to estimate these burdens in this age group is an important contribution of these analyses. This age group, the period of complementary feeding,^10^ is a key target age group for multiple nutrition programmes, although data specific to this period are not reported in global burden statistics. A focus on this age group is critical given that rates of linear growth faltering and wasting are highest during this period.^27^

Some of the top-ranked countries in our analysis also rank highly in the IPC population estimates of acute food insecurity. Prioritising the top-ranked countries is thus in alignment with the WHO guideline recommendations on the use of SQ-LNS in food insecure populations.^10^ The World Bank has also provided recommendations for effective design and implementation of SQ-LNS interventions in the Sahel region to improve child nutrition and health in this region, where high levels of child undernutrition are persistent.^13^ Some of the Sahelian countries such as Niger, Nigeria, Mali, Guinea and Chad were ranked in our analysis as the countries with the highest burden of wasting, stunting and mortality among children aged 6–23 months old. In the identified high burden countries where resources are limited, decisions need to be made about how to prioritise efforts for maximal impact. The recent World Bank Investment Framework for Nutrition listed SQ-LNS as one of the high-priority interventions and part of a cost-effective package of interventions for scalable strategies, informed by an allocative efficiency analysis to identify sets of interventions that can achieve the greatest impact on different outcomes for a fixed budget. The report emphasises that scaling up SQ-LNS requires identifying vulnerable populations and integrating SQ-LNS into existing programmes.^28^ In such programmes, infant and young child nutrition counselling is a necessary component but may not be sufficient by itself when the cost of a nutritionally adequate diet is high, for example, in food insecure regions and when access to nutrient rich food is limited. The results of the decision tree model demonstrate that a large number of children could potentially benefit from provision of SQ-LNS in terms of reduced all-cause mortality, moderate and severe stunting and moderate and severe wasting at the national level. For example, in Niger, which had the highest mortality rate and highest prevalence of severe wasting and severe stunting, provision of SQ-LNS to all children aged 6–23 months could potentially avert over 15 000 cases of severe wasting, over 116 000 cases of severe stunting and over 7000 deaths per year. We recognise that scaling up SQ-LNS in the 20 identified countries will be costly and that 100% coverage of SQ-LNS is not realistic, but by providing a range of values for the coverage, we show the potential number of children’s lives that could be improved and saved in each country given a specific level of SQ-LNS coverage. It should be noted that the estimated relative risk reductions in severe wasting and severe stunting in those models may be underestimates, given that more substantial relative risk reductions were observed in the higher-burden sites in the individual participant data analysis, for example, a 48% reduction in severe wasting in sites with poor water quality.^8^ Therefore, the number of cases of severe wasting and severe stunting potentially averted could be higher than shown herein.

One key limitation of our analysis is that the survey data we used to estimate wasting prevalence in some countries were collected over 10 years ago. Also, we used cross-sectional surveys in our analysis, which underestimates the incidence and total burden of wasting in comparison with longitudinal cohort data.^29 30^ In addition, we assumed that the effect of SQ-LNS on each adverse outcome in our simulation was linear, and we did not account for the potentially nonlinear dynamic interplay between this preventive intervention and wasting and stunting prevalence. Future work is needed to develop a dynamic model of wasting and stunting and to account for the potential nonlinear trade-off between treatment and preventive interventions as well as the cost-effectiveness of provision of SQ-LNS in reducing the treatment demand and overall burden of undernutrition.

## Conclusion

Our analysis identified countries with a high burden of undernutrition and mortality among children 6–23 months old, with the majority located in Sub-Saharan Africa and a few in South and Southeast Asia. These findings highlight the urgent need for targeted interventions in these regions. Scaling up of SQ-LNS, integrated into existing programmes together with other life-saving interventions such as immunisations and included in nutrition responsive social protection systems, could play a critical role in reducing undernutrition and improving child health outcomes. While our analysis is not designed for direct programme decision-making within countries, it provides information to help decision-makers and funders identify priority countries and subregions for SQ-LNS scale-up.

## Supplementary material

10.1136/bmjgh-2025-019353 online supplemental file 1

## Data Availability

Data are available in a public, open access repository.
